# Supplementary material for: Implication of gut microbiota in the physiology of rats intermittently exposed to cold and hypobaric hypoxia
Source: PLoS One. 2020 Nov 3;15(11):e0240686. doi: 10.1371/journal.pone.0240686 (PMC7608931; doi:10.1371/journal.pone.0240686)
Supplement: S1 Table — (DOCX) [file pone.0240686.s001.docx]

**Supporting information**

S1 Table.- Quantitative real-time PCR primers and conditions

| Target bacteria | | Annealing temperature (ºC) | Sequences (5’-3’) | Positive Control | Reference |
| --- | --- | --- | --- | --- | --- |
| Total Bacteria | | 65 | F: ACT CCT ACG GGA GGC AGC AGT | (a) | (33) |
|  | |  | R: ATT ACC GCG GCT GCT GGC |  |  |
| Bacteroidetes | | 62 | F: ACG CTA GCT ACA GGC TTA A | *Bacteroides fragilis* | (34) |
|  | |  | R: ACG CTA CTT GGC TGG TTC A |  |  |
| Firmicutes | | 52 | F: CTG ATG GAG CAA CGC CGC GT | *Ruminococcus productus* | (35) |
|  | |  | R: ACA CYT AGY ACT CAT CGT TT |  |  |
| Bacteroidales | | 61 | F: GGT GTC GGC TTA AGT GCC AT | *Bacteroides fragilis* | (33) |
|  | |  | R: CGG AYG TAA GGG CCG TGC |  |  |
| Clostridiales | | 60 | F: CGG TAC CTG ACT AAG AAG C | *Ruminococcus productus* | (33) |
|  | |  | R: AGT TTY ATT CTT GCG AAC G |  |  |
| Lactobacillales | 60 | | F: AGC AGT AGG GAA TCT TCC A | *Lactobacillus acidophylus* | (36) |
|  |  | | R: CAC CGC TAC ACA TGG AG |  |  |
| Bifidobacteriales | 55 | | F: CTC CTG GAA ACG GGT GG | *Bifidobacterium longum* | (37) |
|  |  | | R: GGT GTT CTT CCC GAT ATC TAC A |  |  |
| Enterobacteriales | 60 | | F: ATG GCT GTC GTC AGC TCG T | *Escherichia coli* M15 | (33) |
|  |  | | R: CCT ACT TCT TTT GCA ACC CAC T |  |  |

^a^ Positive control for total bacteria was the same as that for each individual reaction.
