# Supplementary material for: Implication of gut microbiota in the physiology of rats intermittently exposed to cold and hypobaric hypoxia
Source: PLoS One. 2020 Nov 3;15(11):e0240686. doi: 10.1371/journal.pone.0240686 (PMC7608931; doi:10.1371/journal.pone.0240686)
Supplement: Attachment — Submitted filename: Ramos-Romero_Response to Reviewers.docx [file pone.0240686.s002.docx]

Barcelona, 20^th^ July 2020

Dear Editor and Reviewer,

We would like to thank you for your time and effort concerning our manuscript and the constructive criticism we have obtained. We have addressed the concerns raised by the reviewer in our point-by-point answers and done revisions to the manuscript accordingly as indicated in the following answers.

1. Rats raised in 22 C temperature seems too low, rats in this ambient temperature facing cold exposure and thus can increase the thermogenesis (such as from brown adipose tissue and other organs) to keep stable body temperature, and/or increase the food consumption. For 4 C exposure, this is the further (or additive effect) low temperature effect.

Rats were raised at a standard room temperature of 22 ºC, following the guidelines from European Union and the regulations of the Governments of Catalonia and Spain. Concretely, EU commission recommend rodents should be maintained within a temperature range of 20 ºC to 24 ºC, as follow: <https://eur-lex.europa.eu/legal-content/EN/TXT/?uri=celex:32007H0526>

The rat’s thermo-neutral zone ranges from 27 to 30 ºC (Gordon, Lee, Chen et al 1991), nevertheless the results obtained in the studied groups are referred to the control group, raised at 22 ºC. In this sense, the results obtained from 4 ºC exposure are an additive effect, as the reviewer pointed out. This is now included in the Discussion section (Lines 243-246)

2. For brown adipose tissue, from which location and how many BAT removed from the body? if showed some data for UCP1 in BAT and non-shivering thermogenesis will be benefit for the conclusion.

The brown adipose tissue collected was the whole interscapular BAT from all animals (now indicated in Line 121).

We agree with the reviewer about the interest about UCP1 in BAT and non-shivering thermogenesis in this kind of studies. Unfortunately, we have not the techniques available to analyse that in the present experiment. We will include them in future works.

3. It is better to do some transplanting experiments, such as transplanting gut microbiota of cold rats to 22C rats, or from hypoxia to normal condition, or the combination.

Despite the interest of a transplantation experiment, it is not possible to do it without the pertinent approval from the Ethical Committee for Animal Experimentation. For the present project, we cannot ask for such authorization since faecal transplantation was not included in the aims of the funded project.
